# Supplementary material for: Long-term treatment with senolytic drugs Dasatinib and Quercetin ameliorates age-dependent intervertebral disc degeneration in mice
Source: Nat Commun. 2021 Sep 3;12:5213. doi: 10.1038/s41467-021-25453-2 (PMC8417260; doi:10.1038/s41467-021-25453-2)

Supplementary Information

Long-term treatment with senolytic drugs Dasatinib and Quercetin ameliorates age-dependent intervertebral disc degeneration in mice

Supplemental Figure 1: Successful outcome of D+Q treatment in ameliorating age-dependent intervertebral disc degeneration depends on the baseline degeneration status. (a-b) Representative histology of Veh and D+Q treated mice from 6-23M and 18-23M cohorts, respectively. Representative low (5X) and high (20X) magnification views of a whole disc and NP, AF, and CEP compartments are shown. (**c-d)** Level-by-level analysis of Modified Thompson Grading averages of NP and AF compartments from L3-L6 lumbar discs of 6-23M, and 18-23M cohorts. 6-23M Veh (n=13), D+Q (n=15); 18-23M Veh (n=11), D+Q (n=9); 3 lumbar levels per group were analyzed. Two-tailed t-test or Mann-Whitney test was used as appropriate for comparing differences between Veh and D+Q groups. Data are represented as mean ± SEM. Low magnification scale bar = 200 µm (first column: a, b); high magnification scale bar = 50 µm. NP: nucleus pulposus; AF: annulus fibrosus; EP: endplate. Source data are provided as a Source Data file.

Supplemental Figure 2: Lack of disc cell proliferation following D+Q treatment and status of disc cell senescence burden corelates to outcomes of D+Q treatment regimen. (a-d) Immunostaining and staining area quantifications of cell cycle and senescence markers in the disc compartments of 14-23M cohort: RB, pRB, and pH2AX. **(e-g'')** Immunohistological staining and staining area quantifications of senescence status in the disc compartments from 18-23M cohort: $p16^{INK4a}$, $p19^{ARF}$, RB, and IL-6. Two-tailed t-test or Mann-Whitney test was used as appropriated; n=6 mice/group, 2-3 levels per mouse were analyzed. Data are represented as mean ± SEM. Scale bar =200 µm. Source data are provided as a Source Data file.

Supplemental Figure 3: AF and NP tissues present distinct transcriptomic profiles in aged C57BL/6 mice. (a) Representative percentage of upregulated DEGs in the AF and NP comparison from 23-month-old mice from the 14-23M Veh cohort. **(b-c)** Schematic summarizing the DEGs between AF and NP of 23-month-old mice related to focal adhesion and endochondral bone ossification pathways. Source data are provided as a Source Data file.

Supplemental Figure 4: D+Q treatment promoted unique transcriptomic profiles in the AF and NP compartments. (a) Representative percentage of up- and downregulated DEGs between D+Q and. Veh AF tissues from the 14-23M cohort, $p \leq 0.05$ **(b)** Hierarchical clustering analysis of DEGs between D+Q and Veh AF tissues. **(c)** Representative DEGs from selected GO processes from upregulated DEGs between D+Q and Veh tissues **(d)** Representative DEGs from selected GO processes from downregulated DEGs between D+Q and Veh AF tissues **(e)** Representative percentage of up- and downregulated DEGs between D+Q and Veh NP tissues, $p \leq 0.05$. **(f)** Hierarchical clustering analysis of DEGs between D+Q and Veh NP tissues **(g)** Representative DEGs from selected GO processes from downregulated DEGs between D+Q and Veh. NP tissues. GO process enrichment analysis was performed using the PANTHER Overrepresentation Test with GO Ontology database annotations and a binomial statistical test with FDR $\leq$ 0.05. **(h)** Representative DEGs from selected GO processes from upregulated DEGs between D+Q and Veh. NP tissues **(i)** Representative Top 50 upregulated DEGs between D+Q and Veh NP tissues, $p \leq 0.05$. Source data are provided as a Source Data file.

Supplemental Figure 5: D+Q treatment prevented the age-associated systemic increase in proinflammatory molecules, cytokines, and Th17-related proteins. Multiplex analysis of the **(a-a')** proinflammatory molecules, **(b-b')** cytokines, and **(c-c')** Th17-related proteins in serum from 6-23M Veh (n=12) and D+Q (n=12) and 18-23M Veh (n=9) and D+Q (n=8) cohorts. Two-tailed t-test or Mann-Whitney test was used as appropriate, n=8-12 mice/group. Data are represented as mean ± SEM. Source data are provided as a Source Data file.

Supplemental Figure 6: Long-term D+Q treatment was well tolerated by mice. (a) Survival curve from 6-23M Veh and D+Q cohorts. **(b)** Weight progression in males from 6-23M Veh (n=6) and D+Q (n=7) cohorts. **(c)** Weight progression in females from 6-23M Veh (n=7) and D+Q (n=8) cohorts. **(d)** Survival curve from 18-23M Veh and D+Q cohorts. **(e)** Weight progression in males from 18-23M Veh (n=5) and D+Q (n=5) cohorts. **(f)** Weight progression in females from 18-23M Veh (n=6) and D+Q (n=4) cohorts. **(g)** Grip test comparison of 6-23M Veh (n=15) and D+Q (n=15) cohorts. Data are represented as mean ± SEM. Source data are provided as a Source Data file.

Supplemental Figure 7: Effect of long-term D+Q treatment regimens on disc height and DHI in mice. (a-c) Disc height, vertebral height, and disc height index (DHI) comparisons between **(a)** 6-23M Veh (n=13) and D+Q (n=15), **(b)** 14-23M Veh (n=8) and D+Q (n=7), and **(c)** 18-23M Veh (n=11) and D+Q (n=9) cohorts, L4-L6 /mouse. Two-tailed t-test or Mann-Whitney test was used as appropriate, n=6-13 mice/group. Data are represented as mean ± SEM. Source data are provided as a Source Data file.

Supplemental Figure 8: Effects of D+Q on trabecular bone quality parameters in 6-23M and 18-23M treatment cohort. (a-b) 3D reconstruction showing a transverse section through representative male and female lumbar vertebrae from 6-23M and 18-23M Veh and D+Q cohorts. Scale bar E-F = 500 μm. **(c-f)** Analysis of trabecular bone parameters **(c-c')** BV/TV **(d-d')** trabecular thickness (Tb.Th), **(e-e')** trabecular number (Tb.N), and **(f-f')** trabecular space (Tb.Sp) between Veh and D+Q males and females during aging and in the 6-23M and 18-23M cohorts. Males 6-23M: Veh n=6, D+Q n=7; Females 6-23M: Veh n=7, D+Q n=8. Males 18-23M: Veh n=5, D+Q n=5. Females 18-23M: Veh n=6, D+Q n=4. Vertebrae L4-L6 /mouse. Two-tailed t-test or Mann-Whitney test was used as appropriate. Data are represented as mean ± SEM. Source data are provided as a Source Data file.

Supplemental Figure 9: Effect of D+Q on cortical bone parameters in 6-23M and 18-23M treatment cohort. (a-b) 3D reconstruction showing surface view and hemisection through representative male and female lumbar motion segment from 6-23M and 18-23M cohorts. Scale bar e-f = 500 μm. **(c-f)** Analysis of cortical bone parameters **(c-c')** Bone volume (BV) **(d-d')** bone area (B.Ar) **(e-e')** cortical bone thickness (Cs.Th), and **(f-f')** eccentricity cross-sectional thickness (Ecc) between Veh and D+Q males and females during aging and in the 6-23M and 18-23M cohorts. Males 6-23M: Veh n=6, D+Q n=7; Females 6-23M: Veh n=7, D+Q n=8. Males 18-23M: Veh n=5, D+Q n=5. Females 18-23M: Veh n=6, D+Q n=4. Vertebrae L4-L6 /mouse. Two-tailed t-test or Mann-Whitney test was used as appropriate. Data are represented as mean ± SEM. Source data are provided as a Source Data file.

Supplemental Figure 10: Effect of aging and D+Q treatment on BMD. **(a-d)** Bone mineral density of aged male and female mouse lumbar spines for Veh- and D+Q-treated mice from 6-23M, 14-23M, and 18-23M cohorts. Males 12m: n=5, 23m n=5. Females 12m n=4, 23m n=5. Males

6-23M: Veh n=6, D+Q n=7. Females 6-23M: Veh n=7, D+Q n=8. Males 18-23M: Veh n=5, D+Q n=5. Females 18-23M: Veh n=6, D+Q n=4. Vertebrae L4-L6 /mouse. Two-tailed t-test or Mann-Whitney test was used as appropriate. Data are represented as mean ± SEM. Source data are provided as a Source Data file.

Supplemental Figure 11: Long-term systemic D+Q treatment did not ameliorate age-dependent knee articular cartilage degeneration and senescence burden in mice. (a) OARSI histological score (summed across 4 quadrants, 0-6 score for each) in 6-23M Veh (n=7) and D+Q (n=7) and 18-23M Veh (n=6) and D+Q (n=5) cohorts. **(b)** OARSI grading in males and females in the Veh and DQ groups across all treatment durations analyzed in the study. n=Veh: 12 male, 7 female; DQ: 14 male, 5 female. **(c-e'')** Staining and abundance analysis of key markers of senescence in the knee: $p16^{INK4a}$, $p19^{ARF}$and P21. M: Meniscus. n=5 mice/group. Two-tailed t-test or Mann-Whitney used as appropriate. Data are represented as mean ± SEM. Source data are provided as a Source Data file.

Supplementary Figure 1

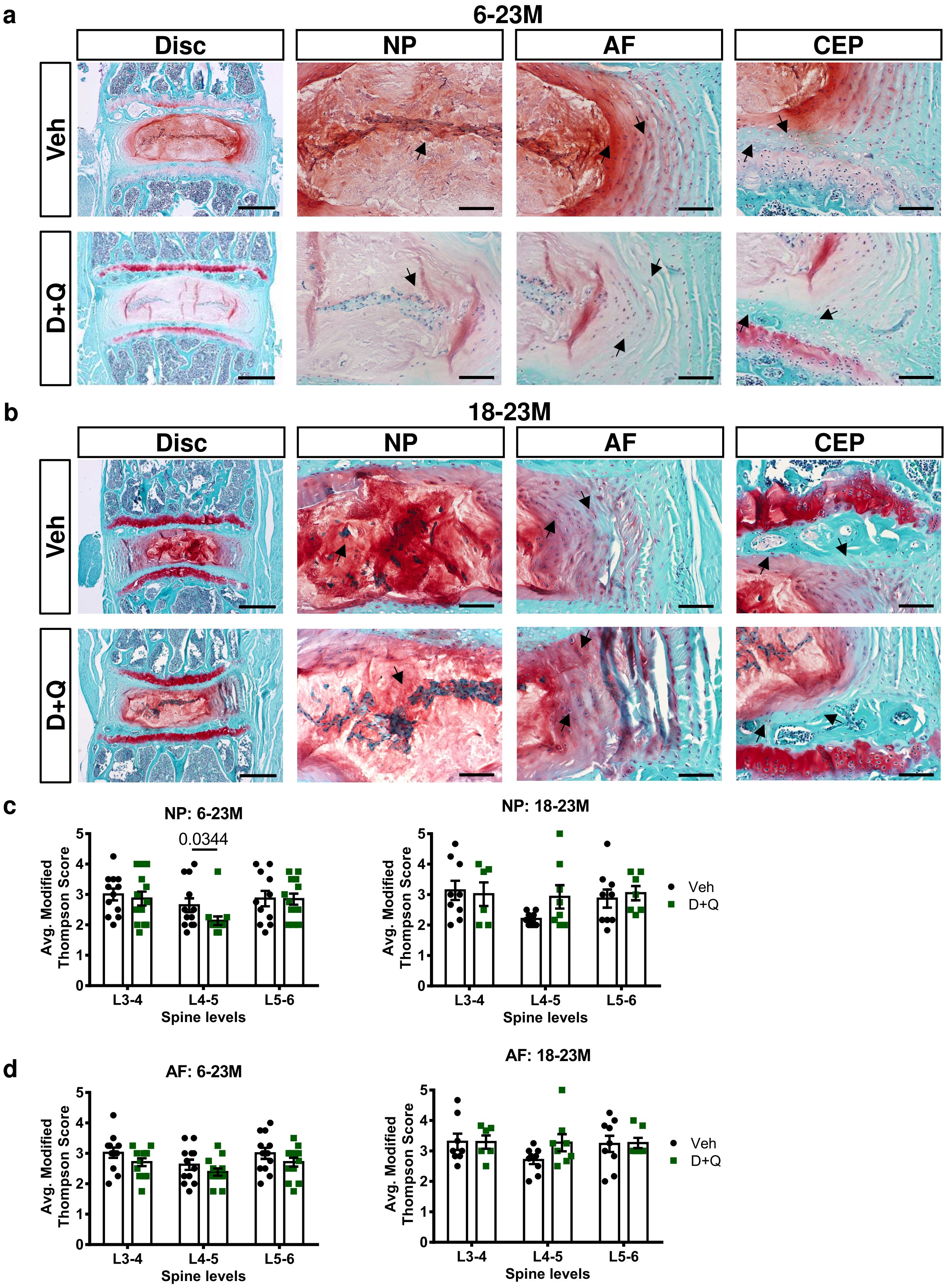

Supplementary Figure 2

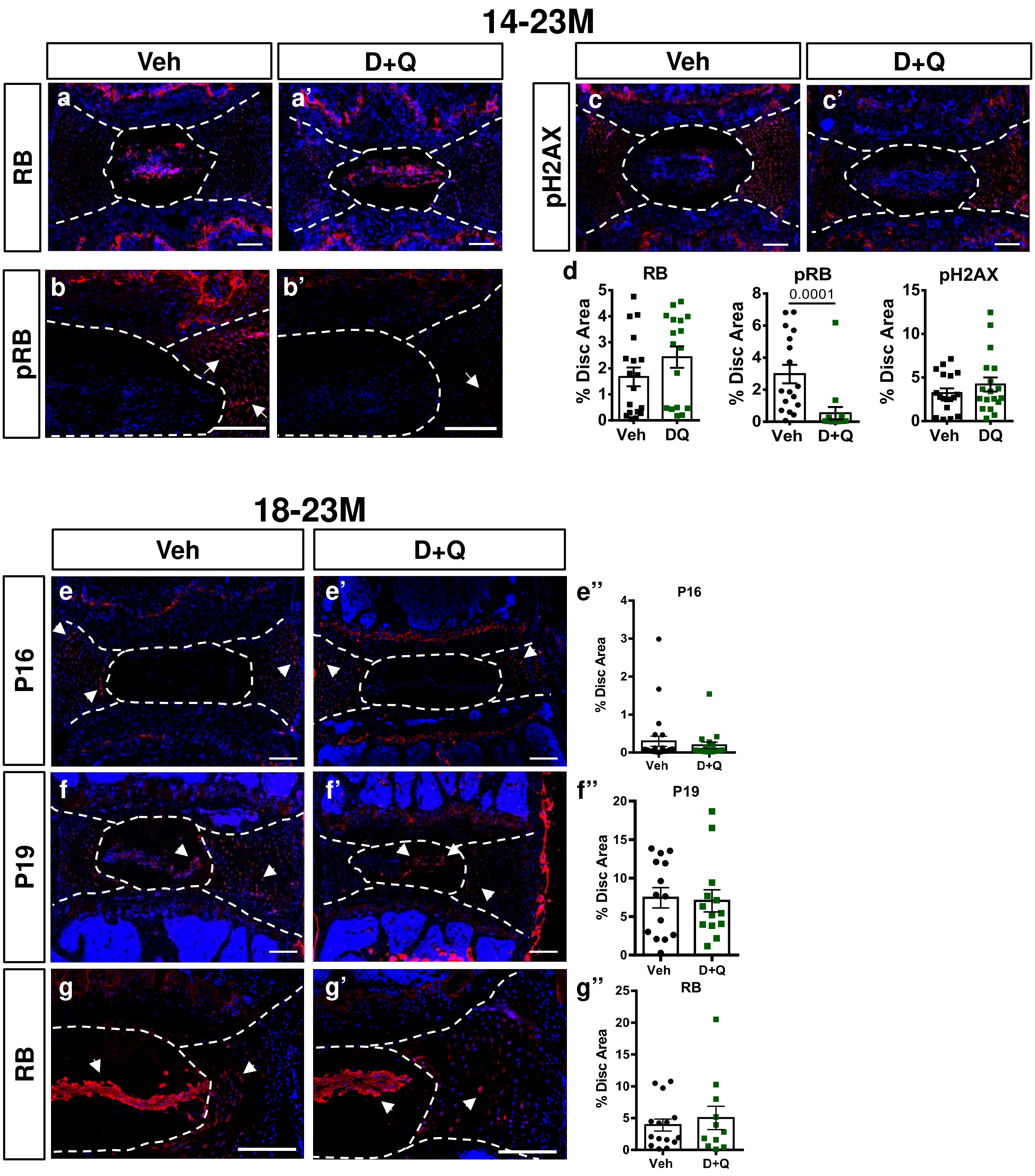

Supplementary Figure 3

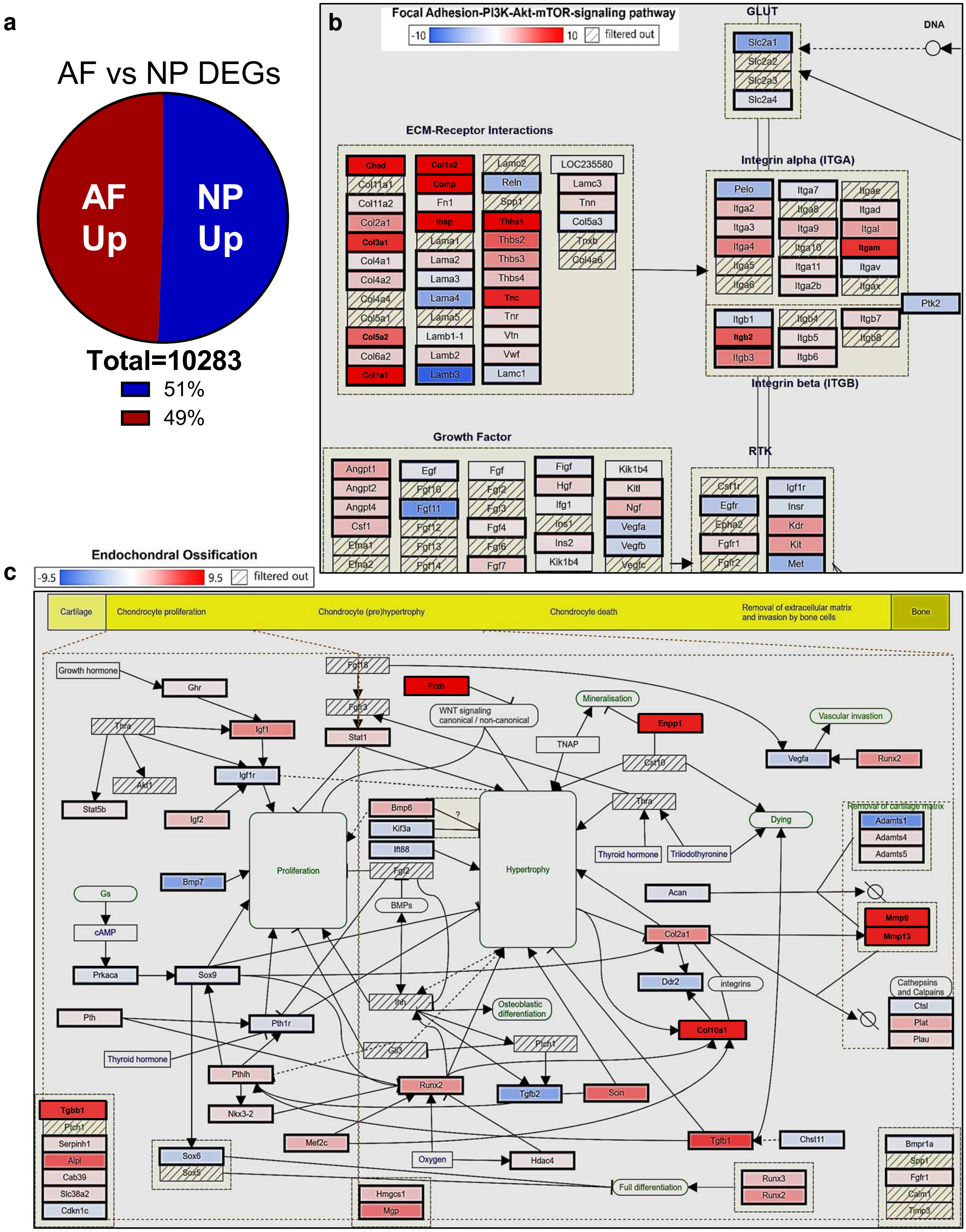

Supplementary Figure 4

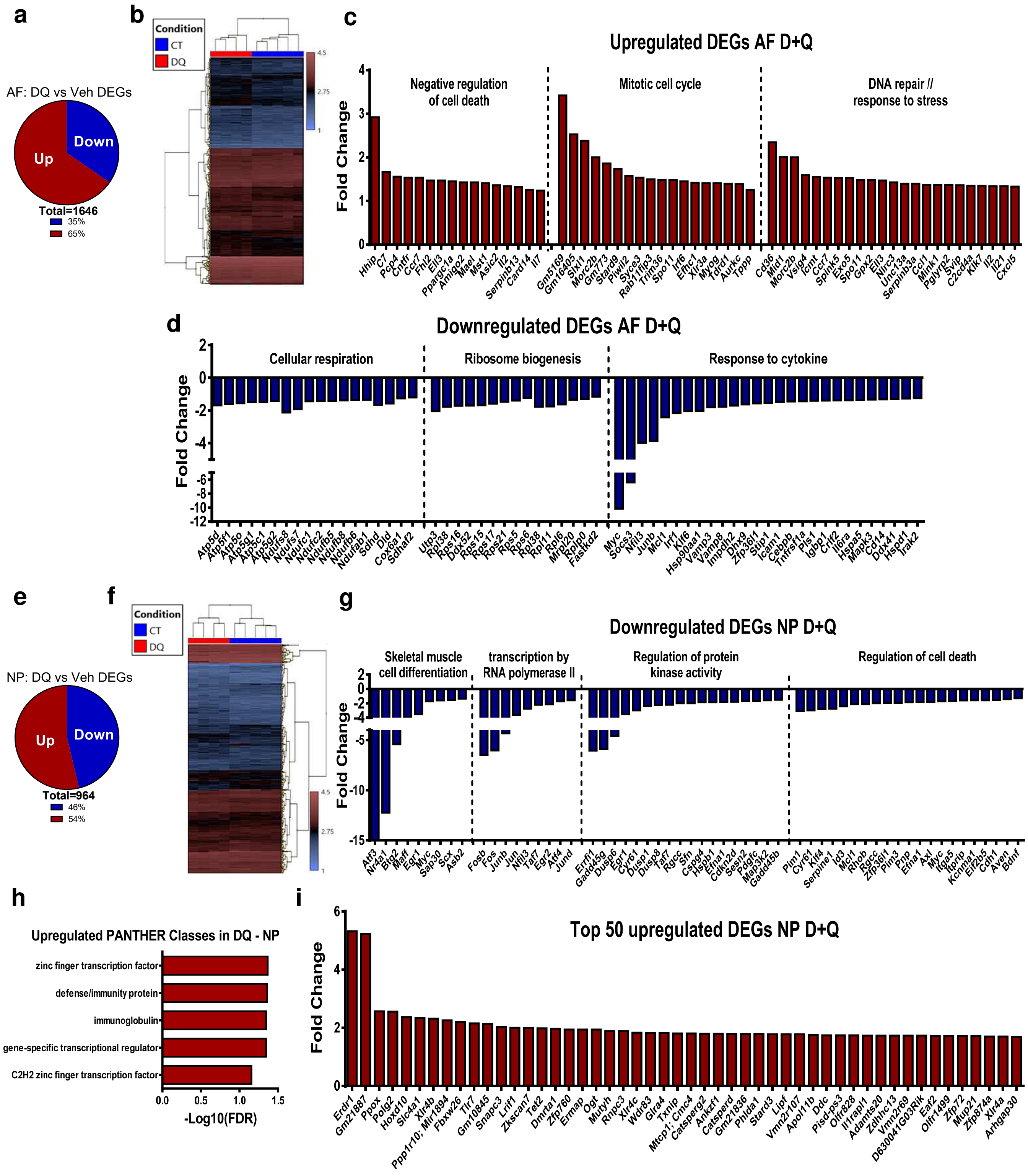

Supplementary Figure 5

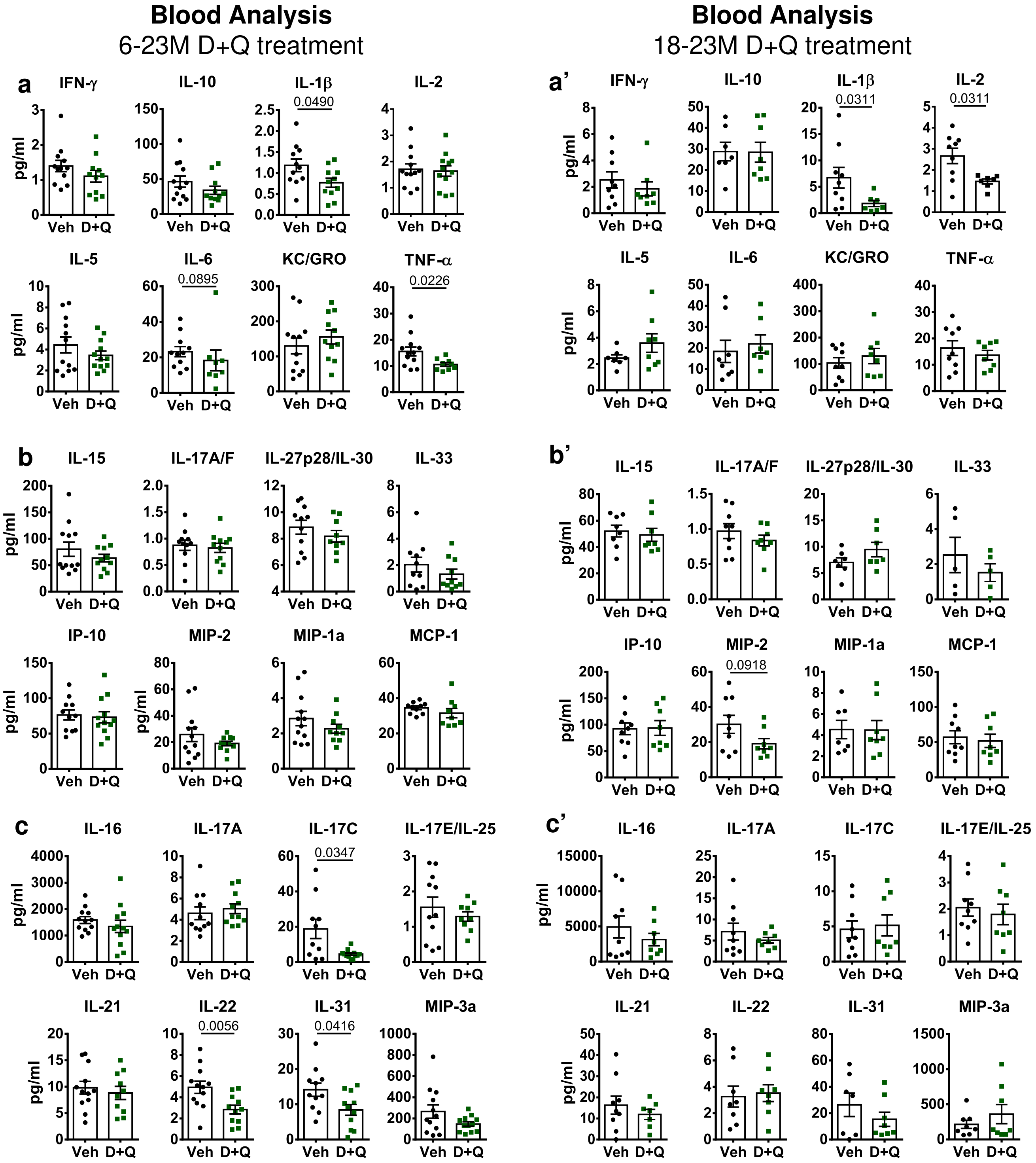

Supplementary Figure 6

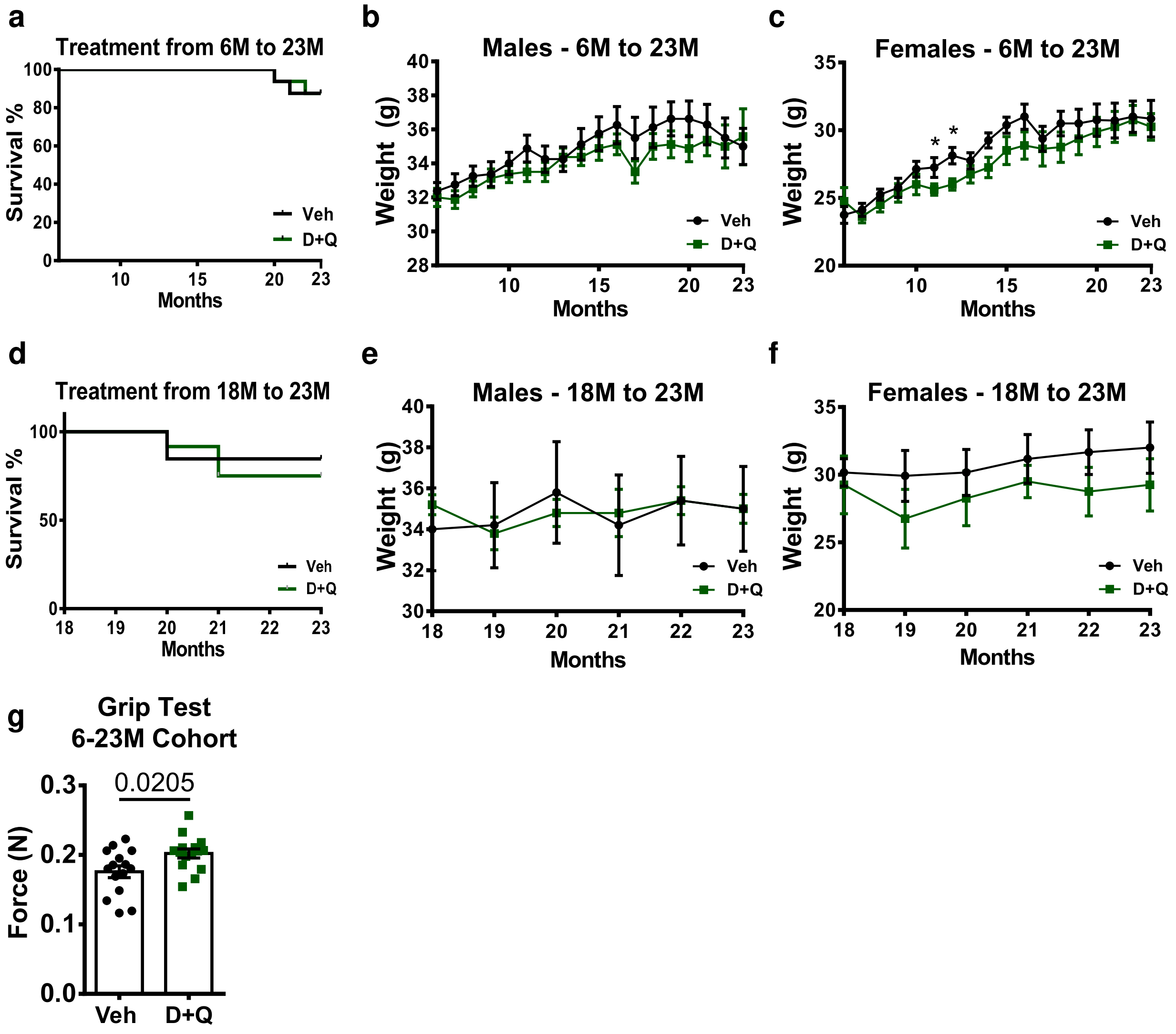

Supplementary Figure 7

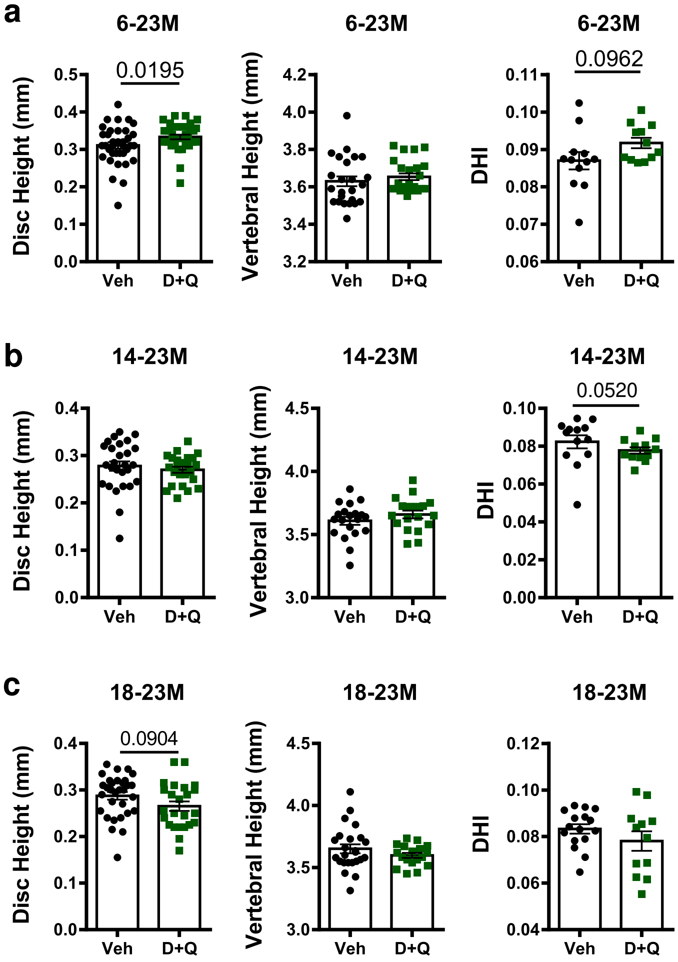

Supplementary Figure 8

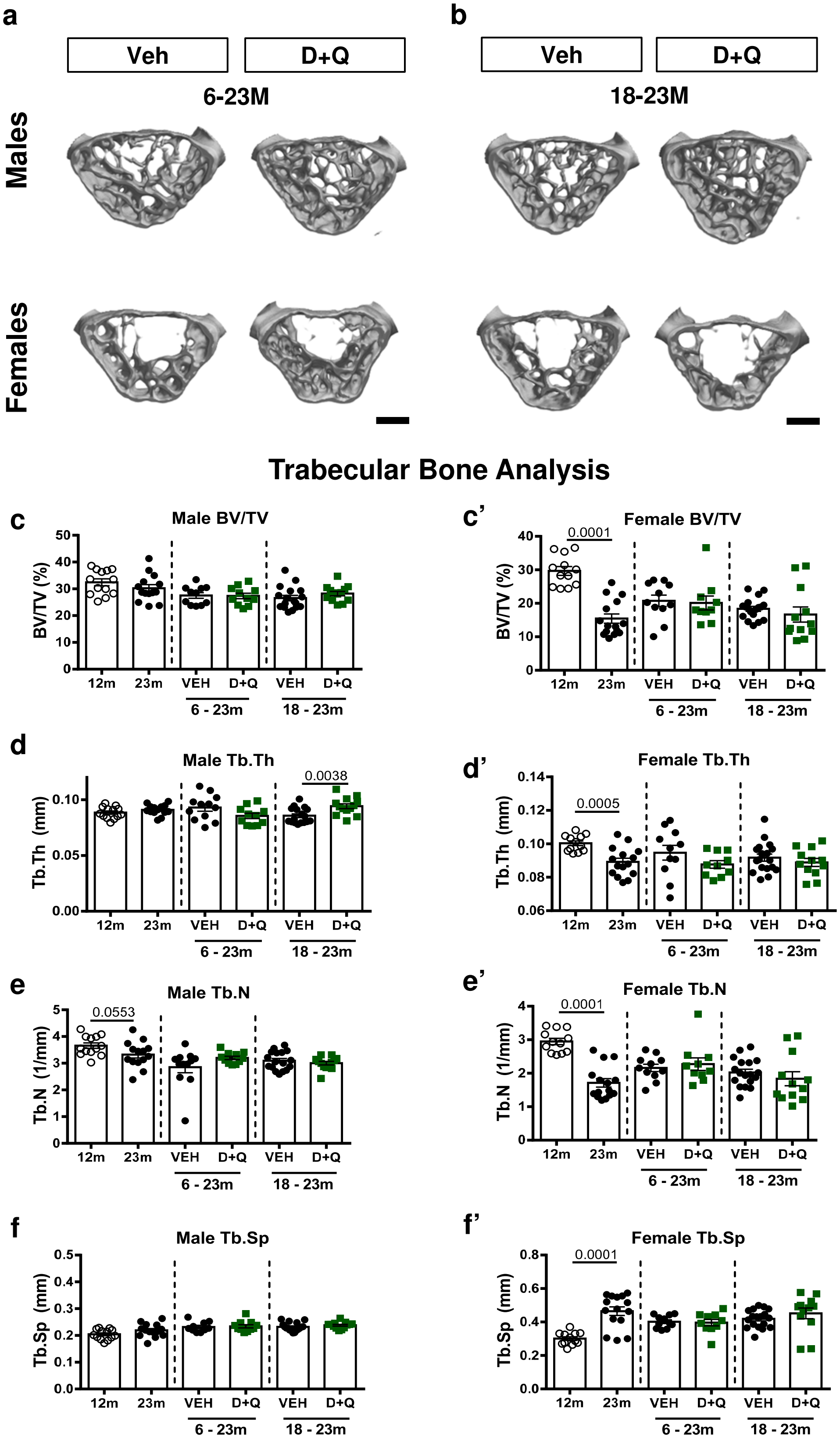

Supplementary Figure 9

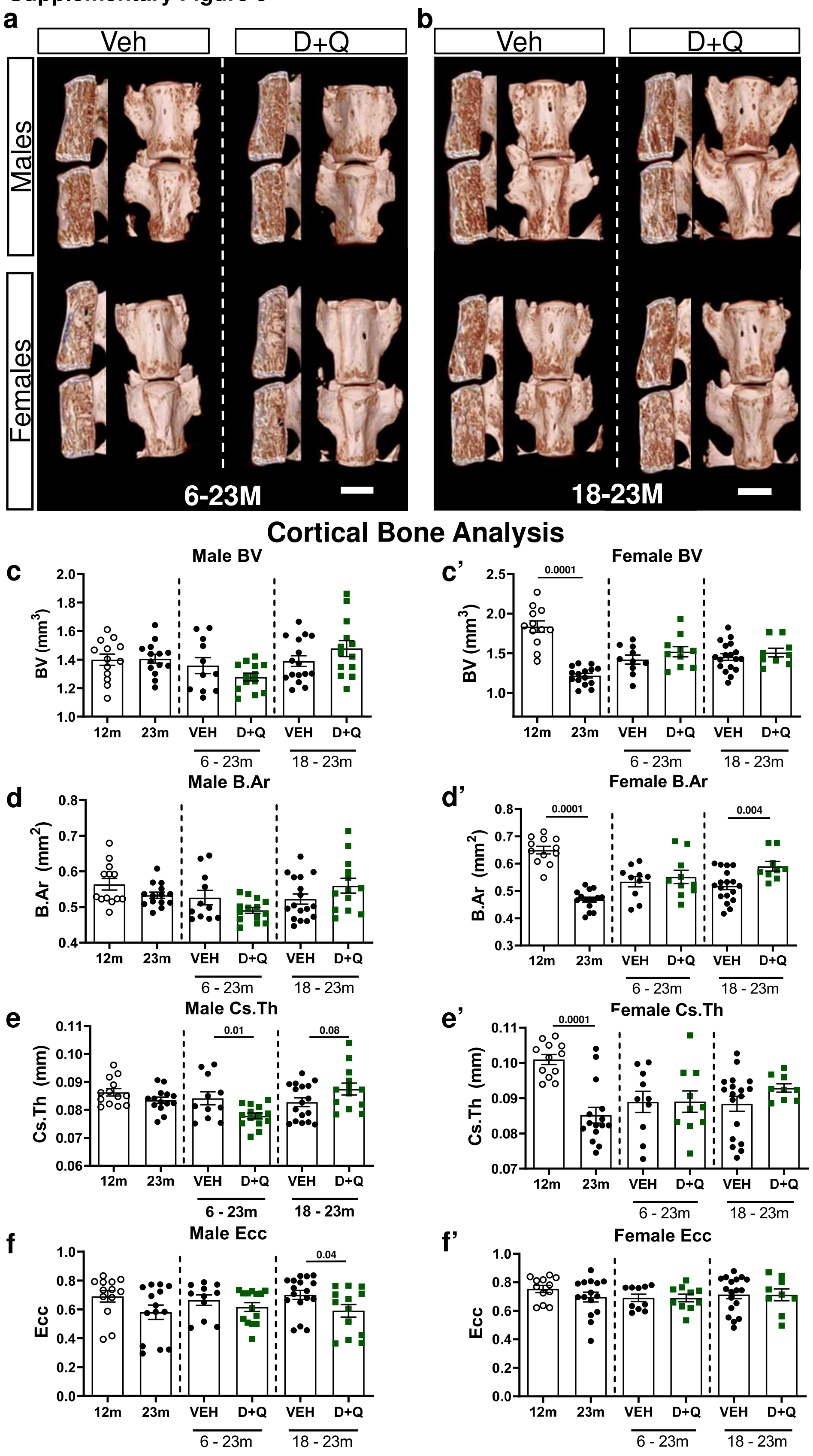

Supplementary Figure 10

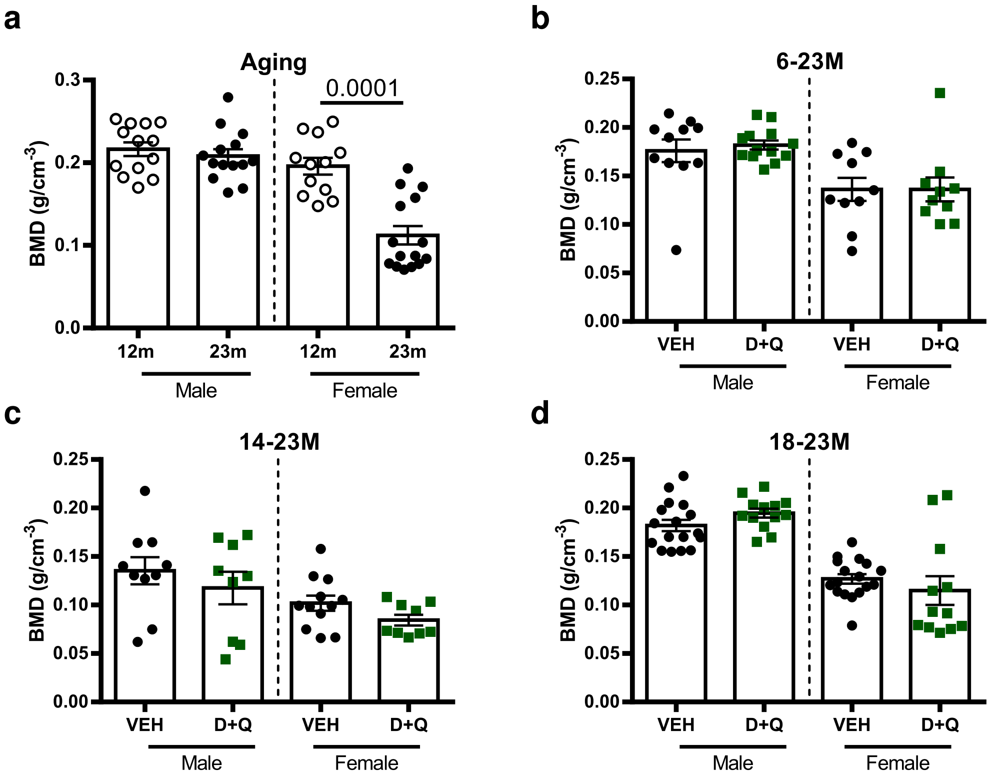

Supplementary Figure 11

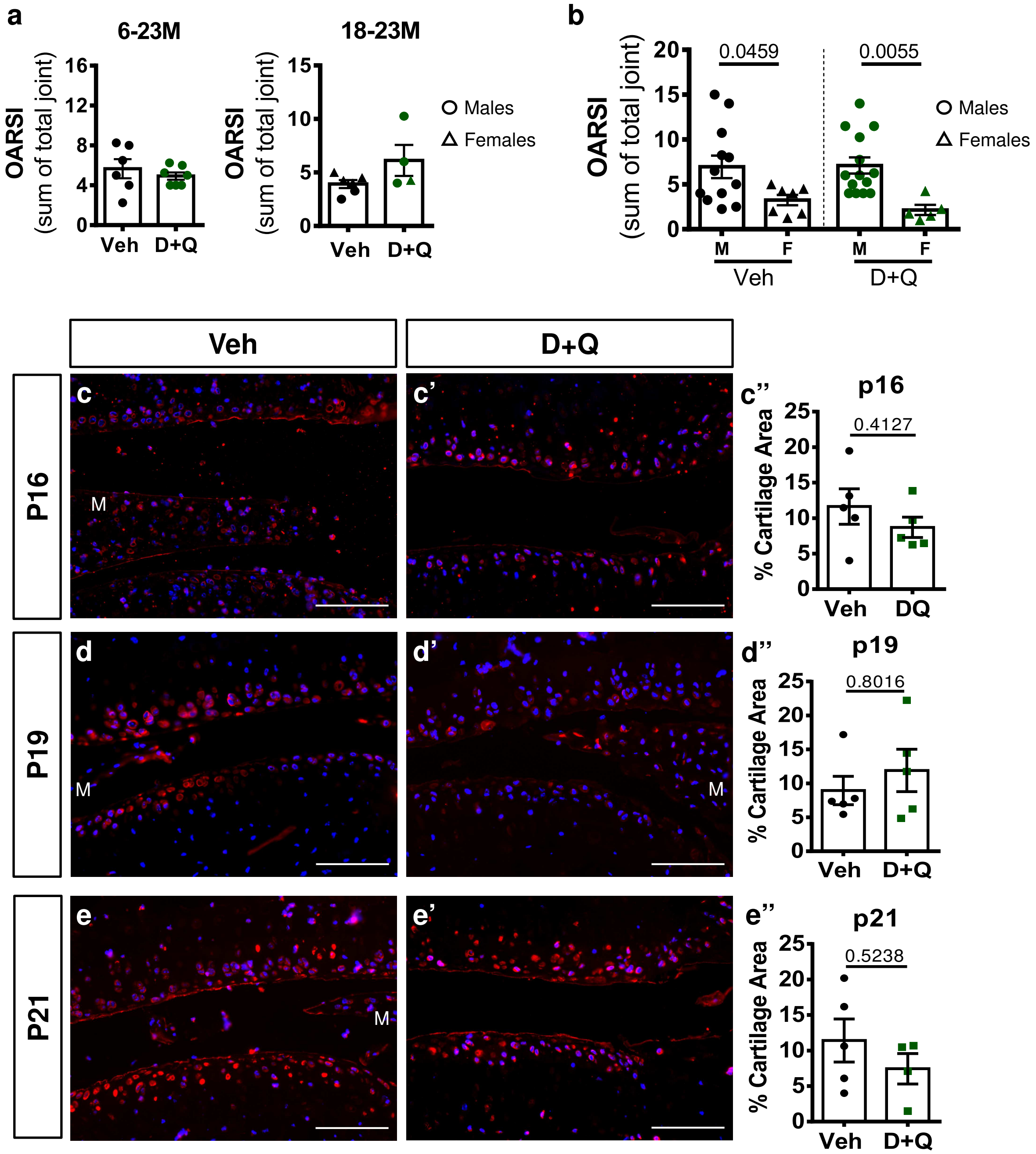

Supplement: Supplementary file 1 — Supplementary Information [file 41467_2021_25453_MOESM1_ESM.pdf]
