# Supplementary material for: Long-term treatment with senolytic drugs Dasatinib and Quercetin ameliorates age-dependent intervertebral disc degeneration in mice
Source: Nat Commun. 2021 Sep 3;12:5213. doi: 10.1038/s41467-021-25453-2 (PMC8417260; doi:10.1038/s41467-021-25453-2)
Supplement: Supplementary file 3 — Reporting Summary [file 41467_2021_25453_MOESM3_ESM.pdf]

nature research

Corresponding author(s): Emanuel J. Novais, Victoria A. Tran, Shira N. Johnston, Kayla R. Darris, Alex J. Roupas, Garrett A. Sessions, Irving M. Shapiro, Brian O. Diekman, Makarand V. Risbud

Last updated by author(s): 2021/07/07

Reporting Summary

Nature Research wishes to improve the reproducibility of the work that we publish. This form provides structure for consistency and transparency in reporting. For further information on Nature Research policies, see our Editorial Policies and the Editorial Policy Checklist.

Statistics

For all statistical analyses, confirm that the following items are present in the figure legend, table legend, main text, or Methods section.

n/a	Confirmed	
☐	☒	The exact sample size (*n*) for each experimental group/condition, given as a discrete number and unit of measurement
☐	☒	A statement on whether measurements were taken from distinct samples or whether the same sample was measured repeatedly
☐	☒	The statistical test(s) used AND whether they are one- or two-sided<br>*Only common tests should be described solely by name; describe more complex techniques in the Methods section.*
☐	☒	A description of all covariates tested
☐	☒	A description of any assumptions or corrections, such as tests of normality and adjustment for multiple comparisons
☐	☒	A full description of the statistical parameters including central tendency (e.g. means) or other basic estimates (e.g. regression coefficient) AND variation (e.g. standard deviation) or associated estimates of uncertainty (e.g. confidence intervals)
☐	☒	For null hypothesis testing, the test statistic (e.g. F, t, r) with confidence intervals, effect sizes, degrees of freedom and P value noted<br>*Give P values as exact values whenever suitable.*
☒	☐	For Bayesian analysis, information on the choice of priors and Markov chain Monte Carlo settings
☐	☒	For hierarchical and complex designs, identification of the appropriate level for tests and full reporting of outcomes
☒	☐	Estimates of effect sizes (e.g. Cohen's *d*, Pearson's *r*), indicating how they were calculated

Our web collection on statistics for biologists contains articles on many of the points above.

Software and code

Policy information about availability of computer code

Data collection: Command Console Software (v.AGCC 4.0, Affymetrix, Santa Clara, CA, USA); Skyscan 1275 version 1.0.16.0; Spectrum Spotlight 400 FT-IR Imaging system (Perkin Elmer, Shelton, CT), nRecon (Bruker) program; Discovery Workbench 4.0; Zen2TM software (Carl Zeiss AG, Germany);

Data analysis: NIS Elements AR 4.50.00 (Nikon, Tokyo, Japan); ISys Chemical Imaging Analysis software (v. 5.0.0.14); Spectrum Image Software; Image J software, v1.53e; Affymetrix Transcriptome Analysis Console (TAC) 4.0 software; CTAn (version 1.17.7.2+ 64bit Bruker microCT); nRecon Version: 1.7.1.0; Prism7 (GraphPad, La Jolla)

For manuscripts utilizing custom algorithms or software that are central to the research but not yet described in published literature, software must be made available to editors and reviewers. We strongly encourage code deposition in a community repository (e.g. GitHub). See the Nature Research guidelines for submitting code & software for further information.

Data

Policy information about availability of data

All manuscripts must include a data availability statement. This statement should provide the following information, where applicable:

- Accession codes, unique identifiers, or web links for publicly available datasets
- A list of figures that have associated raw data
- A description of any restrictions on data availability

The microarray dataset that supports the findings of this study is openly available in the GEO database, accession number GSE154619.

Field-specific reporting

Please select the one below that is the best fit for your research. If you are not sure, read the appropriate sections before making your selection.

☒ Life sciences ☐ Behavioural & social sciences ☐ Ecological, evolutionary & environmental sciences

For a reference copy of the document with all sections, see nature.com/documents/nr-reporting-summary-flat.pdf

Life sciences study design

All studies must disclose on these points even when the disclosure is negative.

Sample size	Sample sizes were not predetermined based on statistical methods, but were chosen according to the standards of the field i.e. previous studies investigating effects of senolytics drugs in mice. Studies have successfully shown the beneficial effect of senolysis in the disc (Patil P., Aging Cell, 2019) and senolytics in ameliorating aging and degenerative processes such as vascular atherosclerosis (Roos C., Aging Cell, 2016), fibrotic pulmonary disease (Schafer M., Nat. Comm., 2016), osteoarthritis (Jeon O., Nat. Med, 2017), aging osteoporosis (Farr J., Nat. Med., 2017), aging physical function and lifespan (Xu M, Nat. Med., 2017), using between 3-8 independent samples. Consequently, based on their means and variation, we used 5-16 independent biological samples for each condition and for the disc and vertebrae evaluation 2-3 levels/ mouse, which generated a sufficient number and statistic power for the effect sizes of interest.
Data exclusions	Just the experimental animals that reached the desired time point (23-months) were included in the analysis.
Replication	Reported results were consistently replicated across experiments with all replicates generating similar results: 6-23M cohorts: 4 independent experiments, with 3-4 animals per group (Veh and D+Q); 14-23M cohorts: 2 independent experiments, with 3-4 animals per group (Veh and D +Q); 18-23M cohorts: 4 independent experiments, with 2-3 animals per group (Veh and D+Q).
Randomization	Randomly selected samples and organisms were allocated into experimental groups.
Blinding	Blinding was used in grip test, invertebrate disc degeneration and knee degeneration scoring systems (modified Thompson grading and OARSI grading) due to their subjective nature. Blinding during collection was not needed since conditions were well controlled. Blinding was also not necessary for computational analysis, because the results are quantitative and did not require subjective judgment or interpretation.

Reporting for specific materials, systems and methods

We require information from authors about some types of materials, experimental systems and methods used in many studies. Here, indicate whether each material, system or method listed is relevant to your study. If you are not sure if a list item applies to your research, read the appropriate section before selecting a response.

Materials & experimental systems

n/a	Involved in the study	
☐	☒	Antibodies
☒	☐	Eukaryotic cell lines
☒	☐	Palaeontology and archaeology
☐	☒	Animals and other organisms
☒	☐	Human research participants
☒	☐	Clinical data
☒	☐	Dual use research of concern

Methods

n/a	Involved in the study	
☒	☐	ChIP-seq
☒	☐	Flow cytometry
☒	☐	MRI-based neuroimaging

Antibodies

Antibodies used	collagen I (1:100, Abcam ab34710), collagen II (1:400, Fitzgerald 70R-CR008), COMP (1:200, Abcam ab231977), collagen X (1:500, Abcam ab58632), chondroitin sulfate (1:300, Abcam ab11570); CA3 (1:150, Santa Cruz), p16 (1:50, Abcam ab211542), p19 (1:100, Novus NB200-106), p21 (1:200, Novus NB100-1941), p-H2AX (1:50, Cell Signaling 9718), RB (1:50, Abcam ab181616), pRB (1:50, Cell Signaling D20B12), Ki67 (1:100, Abcam ab15580), IL-1[illegible] (1:100, Novus NB600-633), IL-6 (1:50, Novus NB600-1131), and MMP13 (1:150, Abcam ab39012). For GLUT-1 (1:200, Abcam, ab40084) and ARGxx (1:200, Abcam, ab3773); Alexa Fluor-594 (Jackson ImmunoResearch Lab, Inc.,1:700).
Validation	collagen I (1:100, Abcam ab34710) –positive control, human collagen. https://www.abcam.com/collagen-i-antibody-ab34710.html?productWallTab=Abreviews&applications=74&species=2<br><br>collagen II (1:400, Fitzgerald 70R-CR008) - https://www.fitzgerald-fii.com/collagen-type-ii-antibody-70r-cr008.html<br>COMP (1:200, Abcam ab231977) – positive control: Recombinant human COMP/Cartilage oligomeric matrix protein; Human and mouse cartilage tissue lysate. IHC-P: Human testis tissue. https://www.abcam.com/compcartilage-oligomeric-matrix-protein-antibody-ab231977.html

collagen X (1:500, Abcam ab58632) – specificity: ab58632 recognizes type X collagen. Exhibits slight cross-reactivity with fibronectin and type II and type IX collagen. Does not cross-react with type I, type III, or type XI collagen. https://www.abcam.com/collagen-x-antibody-ab58632.html

chondroitin sulfate (1:300, Abcam ab11570) – positive control: Bovine mammary gland epithelial (BMGE) cells. https://www.abcam.com/chondroitin-sulfate-antibody-cs-56-ab11570.html

CA3 (1:150, Santa Cruz): Silagi E. et al.,Scientific Reports, 2018. https://www.scbt.com/p/ca-iii-antibody-g-5

p16 (1:50, Abcam ab211542): Positive control: MEF whole cell lysate; His-tagged mouse CDKN2A/p16INK4a recombinant protein, aa1-168. ICC/IF: MEF cells. Flow Cyt: MEF cells. IP: MEF whole cell lysate. Negative control: primary deletion of p16INK4A by Dr. Matthew Wolf (provided in Abcam website). https://www.abcam.com/cdkn2ap16ink4a-antibody-epr20418-ab211542.html?productWallTab=ShowAll

p19 (1:100, Novus NB200-106). Tested by western blot and IHC in NIH/3T3 and MEF cells. https://www.novusbio.com/products/p19arf-cdkn2a-antibody_nb200-106
p21 (1:200, Novus NB100-1941). https://www.novusbio.com/products/p21-cip1-cdkn1a-antibody_nb100-1941#reviews-publications

p-H2AX (1:50, Cell Signaling 9718). Positive control: immunohistochemical analysis of paraffin-embedded HT-29 cells untreated (left) or UV-treated (right), using Phospho-Histone H2A.X (Ser139) (20E3) Rabbit mAb. https://www.cellsignal.com/products/primary-antibodies/phospho-histone-h2a-x-ser139-20e3-rabbit-mab/9718

RB (1:50, Abcam ab181616). Validated using knockout cell line and positive control: WB: Jurkat, Hek294, K562, WEHI-3, COS-1, MCF7 and F9 whole cell lysates; Mouse brain and lung lysates; Human fetal brain lysate. IHC-P: Human lung, Human breast cancer, Mouse lung and Mouse cerebral cortex tissues. ICC/IF: MCF7 cells. IP: MCF7 whole cell lysate. https://www.abcam.com/rb-antibody-epr17512-ab181616.html

pRB (1:50, Cell Signaling D20B12). Positive control: immunohistochemical analysis of paraffin-embedded human colon carcinoma and Western blot analysis of extracts from MCF7 cells, untreated (-) or treated with calf intestinal phosphatase (CIP) and λ phosphatase (+). https://www.cellsignal.com/products/primary-antibodies/phospho-rb-ser807-811-d20b12-xp-rabbit-mab/8516

Ki67 (1:100, Abcam ab15580). Validated using knockout cell line. https://www.abcam.com/ki67-antibody-ab15580.html

IL-1β (1:100, Novus NB600-633). Specificity: Detects IL-1 beta and does not cross react with IL-1 alpha. Weakly detects the non-denatured 31 kDa precursor molecule but strongly detects all of the 17 kDa mature molecule. Additionally this IL-1 beta antibody will bind the denatured 31 kDa precursor molecule with greater affinity than the native form, but will often detect past the 35 kDa mark. This IL-1 beta antibody has also been shown to be useful in neutralization of human and primate IL-1 beta activity in bioassays. https://www.novusbio.com/products/il-1-beta-il-1f2-antibody_nb600-633.

IL-6 (1:50, Novus NB600-1131). Specificity: This antiserum detects recombinant and native IL-6 present in body fluids and cell supernatants in various assays (ie. IL-1 stimulated IL-6 production from fibroblasts). In Western blot analysis of natural cell products or human body fluids, multiple bands of IL-6 will appear due to the variable amount of glycosylation on the molecule. The antiserum is also useful for neutralization of human of IL-6 activity in bioassays. For neutralization, incubate the sample with a 1:400 dilution of the antiserum for at least 4 hours before being tested. A control of similarly diluted normal rabbit IgG (heat inactivated) is recommended. In neutralization experiments in vitro, this antibody does not result in enhanced activity of IL-6. However, because antibodies to IL-6 may act as a soluble receptor in vivo, some antibodies to IL-6 act as carriers and enhance IL-6 activity. Publications tested in 4 confirmed species: Human, Mouse, Rat, Monkey, and 7 applications: ELISA, ICC/IF, IHC, IHC-Fr, IHC-P, KD, WB. https://www.novusbio.com/products/il-6-antibody_nb600-1131#datasheet

MMP13 (1:150, Abcam ab39012). Specificity: ab39012 recognizes the latent proenzyme, at 60 Kd, as well as the active form at 48 Kd, and intermediate activation forms. It does not cross react with the other MMP family members. ab39012 recognizes the Hinge region of MMP13. https://www.abcam.com/mmp13-antibody-ab39012.html

GLUT-1 (1:200, Abcam, ab40084). Positive control: HepG2 cells. Esophagous and breast carcinoma. https://www.abcam.com/glucose-transporter-glut1-antibody-spm498-ab40084.html

ARGxx (1:200, Abcam, ab3773): Specificity: Recognises the aggrecanase (ADAMTS-1, -4 & -5)-generated N-terminal neoepitope ARG after cleavage between amino acids EGE and ARG within the interglobular domain of aggrecanase-catabolised aggrecan (Human aggrecan sequence enumeration). This antibody will not recognise the sequence. ARG.. if it is in the non-cleaved intact aggrecan protein core; i.e. it will only recognise the aggrecanase generated neoepitope ARG. https://www.abcam.com/aggrecan-argxx-antibody-bc-3-ab3773.html.

Animals and other organisms

Policy information about studies involving animals; ARRIVE guidelines recommended for reporting animal research

Laboratory animals	6-month-old (15 Females + 13 Males), 14-month-old (7 Females + 8 Males), and 18-month-old (7 Females + 13 Males) C57BL/6 mice were obtained from National Institutes of Aging (NIA) aged rodent colony located at Charles River Laboratories, and aged to 23 months, and analyzed.
Wild animals	No wild animals were used in the study.
Field-collected samples	No field collected samples were used in the study.

Ethics oversight	All animal experiments were performed under IACUC protocols approved by the Thomas Jefferson University.

Note that full information on the approval of the study protocol must also be provided in the manuscript.
